# Optically induced metastability in Cu(In,Ga)Se_2_

**DOI:** 10.1038/s41598-017-14344-6

**Published:** 2017-10-23

**Authors:** S. A. Jensen, A. Kanevce, L. M. Mansfield, S. Glynn, S. Lany, D. Kuciauskas

**Affiliations:** National Renewable Energy Laboratory, 15013 Denver West Pkwy., Golden, Colorado, 80401 USA

## Abstract

Cu(In,Ga)Se_2_ (CIGS) is presently the most efficient thin-film photovoltaic technology with efficiencies exceeding 22%. An important factor impacting the efficiency is metastability, where material changes occur over timescales of up to weeks during light exposure. A previously proposed (*V*_*Se*_ *-V*_*Cu*_) divacancy model presents a widely accepted explanation. We present experimental evidence for the optically induced metastability transition and expand the divacancy model with first-principles calculations. Using photoluminescence excitation spectroscopy, we identify a sub-bandgap optical transition that severely deteriorates the carrier lifetime. This is in accordance with the expanded divacancy model, which predicts that states below the conduction band are responsible for the metastability change. We determine the density–capture cross-section product of the induced lifetime-limiting states and evaluate their impact on device performance. The experimental and theoretical findings presented can allow assessment of metastability characteristics of leading thin-film photovoltaic technologies.

## Introduction

The thin-film photovoltaic (PV) material Cu(In,Ga)Se_2_ (CIGS) has recently achieved a power conversion efficiency of 22.6% for small-area solar cells^1^. This value exceeds that of multicrystalline silicon (21.3%) and is comparable with CdTe and perovskite polycrystalline material systems (both at 22.1%)^2^. Defect analysis and characterization can help understand the processes limiting the efficiency and help further increase the performance and reliability of CIGS solar cells. An important issue is the metastability, where semi-persistent material changes occur over wide-ranging timescales. Metastability effects can have both positive and negative influences on the CIGS solar cell performance. The most common observations are the persistent photoconductivity^3,4^—where the photoconductivity decays slowly after illumination is turned off—and the light-soaking effects^5–7^. Light-soaking effects might differ depending on the irradiation conditions. For example, Rau *et al*. observed increased open-circuit voltage (*V*_*oc*_) and fill factor (*FF)* upon light soaking with white light^8,9^, whereas Heath *et al*. observed a drop in *V*_*oc*_ and *FF* and an increase in the carrier density and trap density in the bulk upon near-bandgap 1064 nm light soaking^10,11^. Different defect states might be affected depending on the light-soaking conditions. Possibilities include defects in the bulk vs. the interfaces and/or defects at different energies within the bandgap. The electronic defect levels can be coupled with lattice relaxation. Lany and Zunger proposed that a divacancy complex (*V*_*Se*_ *-V*_*Cu*_) can transform between two atomic conformations, giving rise to metastable electronic properties^12^. They suggested that electrical bias or illumination can shift this complex from a donor into an acceptor configuration in *p*-type CIGS, increasing the hole concentration, but also creating a potential recombination channel for the minority carriers (electrons).

To study light-induced metastability effects in CIGS polycrystalline films, we employ optical photoluminescence spectroscopy with tunable sub-bandgap energy excitation. Using this approach, we aim to directly excite specific defect states. Optical studies do not require contacts and can be carried out on polycrystalline films, which potentially simplifies data analysis and interpretation. We apply this analysis to CIGS films with band gaps of *E*_*g*_ = 1.10–1.12 eV^13^ and *E*_*g*_ = 1.50 eV, respectively. For the low-*E*_*g*_ samples we also investigated the effect of potassium fluoride (KF) post-deposition treatment, because such treatment is applied in the record-efficiency solar cells^1^. The influence of the defects detected with excitation spectroscopy on device performance is evaluated by numerical simulations using technology computer-aided-design (TCAD) models developed for high-efficiency CIGS solar cells. In this work, we include the energetic distribution of defect bands determined by theory and spectroscopic measurements into the device model, and quantify the sensitivity of a device’s *V*_*oc*_ and efficiency to the defect density.

## Photoluminescence excitation spectroscopy studies

**Figure 1:**
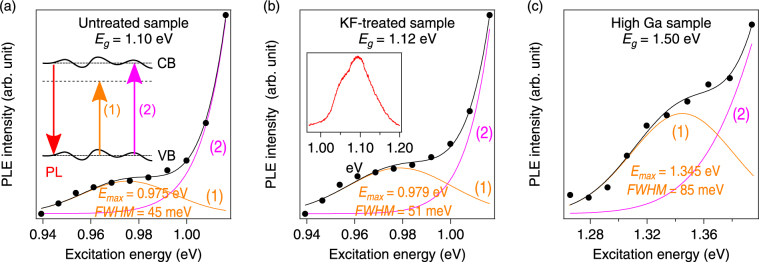
Integrated PL emission intensity as a function of excitation photon energy for three CIGS samples: (**a**) low-*E*_*g*_, (**b**) low-*E*_*g*_ treated with KF, and (**c**) high-*E*_*g*_. Inset in (**a**) shows the model of states (1) and (2) used to fit the sub-bandgap excitation spectroscopy data. Inset in (**b**) shows a PL emission spectrum measured at 300 K for the KF-treated low-*E*_*g*_ sample. Solid lines in (**a**)–(**c**) show fits to the data. Pink curves indicate fits to the near-bandgap PL tails described in ref.^13^, corresponding to process (2) in the inset in (**a**), and orange curves show Gaussian fits to the sub-bandgap feature, process (1). Solid black lines show the sum of Gaussians (1) and (2). The temperature was 300 K.

We observed photoluminescence (PL) emission (at energies corresponding to band-to-band recombination^13^) when the excitation photon energy was well below the bandgap (*E*_*g*_) of the CIGS absorbers. Therefore, the likely mechanism is carrier generation from the valence band (VB) into defect states near the conduction band (CB), as illustrated in the inset of Fig. 1(a). To characterize the defect states, we measured the sub-bandgap excitation spectra shown in Fig. 1. These data were measured by integrating PL emission spectra measured with different excitation wavelengths. Within each excitation spectrum the photon flux at different excitation wavelengths was kept the same and the integration time was constant. The excitation spectra (solid black circles) were fit using a model of two Gaussians, and the black solid lines in Fig. 1 show the sum of the components (labeled (1) and (2) in the inset in Fig. 1(a)). The spectrum of the higher-energy Gaussian component (2) rises with a slope determined by the magnitude of the band-edge potential fluctuations^13^. In addition, we observe a broad spectrum up to 200 meV below the *E*_*g*_, labeled (1). Such spectral features cannot be attributed to band tails, potential, or electrostatic fluctuations, and we ascribe them to a defect band. We determined the energetic distance from the band edge to be 125–155 meV (these numbers are similar for both the low-*E*_*g*_ and high-*E*_*g*_ samples). The full width at half maximum (FWHM) of the defect band was found to be 45–50 meV for the low-*E*_*g*_ samples (Fig. 1(a) and (b)) and FWHM = 85 meV for the high-*E*_*g*_ sample (Fig. 1 (c)). The similar energy of the defect band in the low-*E*_*g*_ and high-*E*_*g*_ samples suggests that the defects observed with excitation spectroscopy are not directly related to the Ga-composition, which is varied to change the band gap in the CIGS absorbers. Similarly, the untreated and KF-treated samples, Fig. 1(a) and (b) respectively, showed very similar behavior, suggesting that the observed defect states were unaffected by the KF-treatment.

The excitation spectra are very different from the emission spectra in^13^, where only the band tails were observed. Deep defects similar to those in Fig. 1 have been observed with PL emission spectroscopy in epitaxially grown ternary CuInSe_2_ and CuGaSe_2_ absorbers, but not for quaternary CIGS compositions^14^. This comparison suggests that with the direct excitation of the defect states it is possible to obtain more detailed defect spectral characteristics in materials where compositional inhomogeneties create electronic band tails and fluctuations.

## First-principles calculations

**Figure 2:**
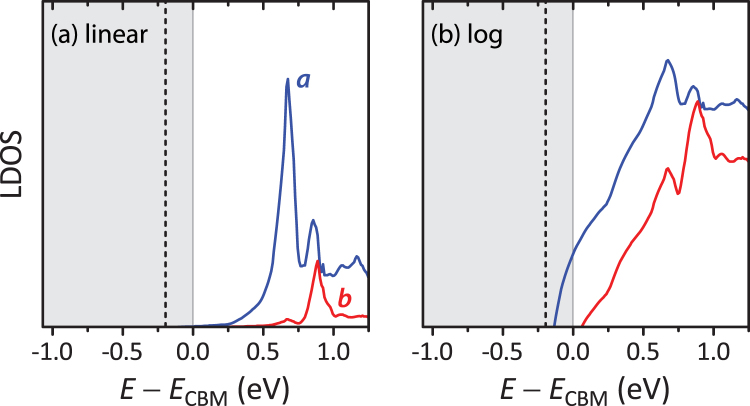
(**a**) Local density of states induced by the *V*_Se_-*V*_Cu_ divacancy pair in CuInSe_2_. The blue and red lines show the *s*- and *p*-like contributions within an empty sphere, respectively, corresponding to the *a* and *b* symmetries (bonding and anti-bonding states) of the defect. The shaded area marks the bandgap of the defect-free matrix, and the vertical dashed line marks the energy of the lowest unoccupied state in the defect system. (**b**) Same data on log scale, highlighting the sub-gap DOS created by the hybridization between the defect and surrounding conduction band states.

We here revisit the divacancy defect model of ref.^12^, using bandgap-corrected GW calculations. Within the present approach (see ref.^15^ for details), the bandgap of CuInSe_2_ is obtained as 1.05 eV. According to ref.^12^, the defect states induced by the divacancy result from formation of a bonding (*a* symmetry) and an antibonding (*b* symmetry) state of the dangling bonds of the two In atoms neighboring the vacant Se site. In *p*-type CuInSe_2_, the divacancy assumes the positive charge state in equilibrium. For this state, Fig. 2 shows the local density of states (DOS) obtained by projection onto an empty sphere centered between the In neighbors. Due to its energetic position as a resonance around 0.7 eV above the conduction band minimum (CBM), the defect states hybridize with conduction band states originating from surrounding atoms. This hybridization creates an energy distribution reaching as far as 0.20 eV into the bandgap, much deeper than the binding energy expected for a perfectly shallow effective mass donor (typically less than 10 meV). Thus, we conclude that the divacancy defect could explain the observed sub-bandgap absorption.

## Time-resolved photoluminescence studies

**Figure 3:**
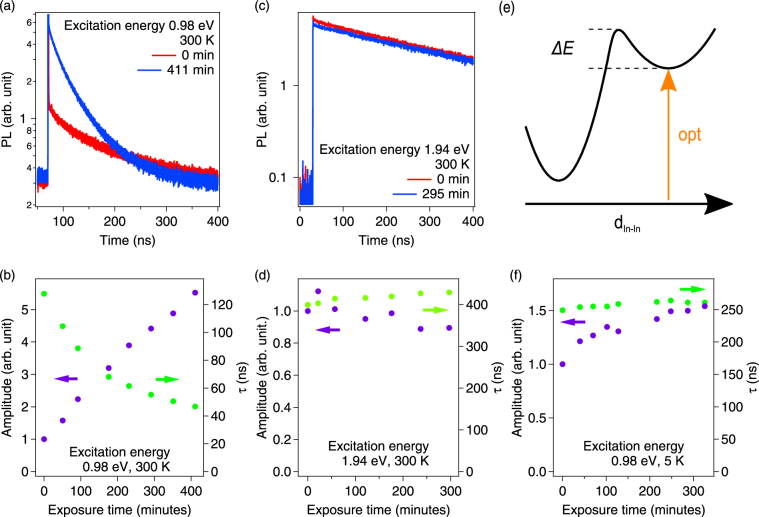
(**a**) TRPL decays measured on the KF-treated E_g_ = 1.12 eV sample immediately and after 411 min of exposure to sub-bandgap light (excitation at 0.98 eV, average power 45 mW) at room temperature. (**b**) Amplitudes and lifetimes from single-exponential fits as a function of irradiation time (room temperature, excitation at 0.98 eV, average power 45 mW). (**c**) TRPL decays measured with above-bandgap photons (room temperature, excitation at 1.94 eV, average power 15 µW) immediately and after 295 min of exposure. (**d**) Amplitudes and lifetimes extracted from single-exponential fits as a function of irradiation time (room temperature, excitation at 1.94 eV, average power 15 µW). (**e**) Illustration of the metastability transition, adapted from ref.^12^. (**f**) Amplitude and lifetime data for exposure to 0.98 eV, 45 mW excitation at 5 K vs exposure time.

To analyze the contributions of the defect states to carrier recombination, we performed TRPL experiments. We first discuss TRPL data measured at 300 K on the *E*_*g*_ = 1.12 eV KF-treated CIGS thin film with 0.98 eV excitation, corresponding to transition (1) in Fig. 1. Figure 3(a) shows a TRPL trace recorded immediately after the sample was exposed to the sub-bandgap light, and a trace recorded after almost 7 h of exposure. The PL intensity with 45 mW excitation at 0.98 eV was found to be similar to the PL intensity with 0.02 mW excitation at 1.94 eV, which was shown to correspond to low-injection conditions^13^. The absorption coefficient at 1.94 eV is 8.7 × 10^4^ cm^−1^ ^16^, and comparison suggests that the absorption coefficient at 0.98 eV is ~50 cm^−1^. Figure 3(b) shows initial PL amplitudes and decay times (extracted from single-exponential fits^17^) vs time of light exposure. It is clear that the sample’s characteristics change dramatically during exposure to 0.98 eV irradiation; an increase in the initial PL amplitude of 500% and a simultaneous decrease in PL lifetime from 128 ns to 47 ns are evident. The PL intensity in the *p*-type absorber is proportional to *B* × *N*_*A*_ × *n*, where *B* is the radiative recombination coefficient, *N*_*A*_ is the net acceptor concentration, and *n* is the minority-carrier concentration generated during the excitation. Assuming that *B* is constant, the amplitude data in Fig. 3(b) directly indicates an increased *N*_*A*_ due to exposure to 0.98 eV irradiation. For comparison, Fig. 3(c) and (d) show that the same sample displayed much smaller metastability with 1.94 eV irradiation at 300 K. After 5 h of irradiation, the changes in amplitude and the minority-carrier lifetime were <20%.

Next, we observed that at low temperature (5 K), the light-induced metastability was strongly reduced. Figure 3(f) shows TRPL lifetimes and amplitudes recorded with 0.98 eV irradiation at 5 K. The data suggest that at low temperature, the thermal energy is not sufficient for interconversion between the metastable defect states as illustrated in Fig. 3(e).

## Discussion and device modeling

In summary, we find much stronger light-induced metastability changes after irradiation at 0.98 eV than with irradiation at 1.94 eV. The spectra of metastable defect states were identified with PL excitation spectroscopy (Fig. 1). The observations presented above are consistent with the model of Lany and Zunger^12^, where light-induced metastability converts the (*V*_*Se*_ *-V*_*Cu*_) divacancy complex into a shallow acceptor. This would increase the net acceptor concentration (*N*_*A*_); therefore the initial PL amplitude would be higher, consistent with the data in Fig. 3(a) and (b). The data suggest an increase in *N*_*A*_ of about 5× (from *N*_*A*_ = 2 × 10^16^ cm^−3^, see ref.^13^, to *N*_*A*_ = 1 × 10^17^ cm^−3^) for 0.98 eV irradiation at 300 K, 1.5× for 0.98 eV irradiation at 5 K, and a slight decrease (by less than 20%) for 1.94 eV irradiation at 300 K. Within the divacancy model, the temperature dependence is due to the energy barriers associated with the electron capture triggering the metastable transition^12^. The present finding of defect states below the CBM (Fig. 2) also provides an explanation for the larger metastable response for sub-gap excitation: When electrons are photoexcited into these states, they could remain locally bound in the vicinity of the defect, and therefore be more readily available for electron capture than the unbound electrons created by above-gap excitation (cf. Fig. 1(a)).

According to the divacancy defect model^12^, the defect assumes a negative state after the metastable transition, in which it creates an additional state about 0.8 eV above the valence band, which can capture minority carriers and act as a recombination center. Such a doubly negatively charged divacancy state was also found in bandgap-corrected hybrid functional calculations^18^. A defect state at similar energies has previously been observed experimentally with transient photocapacitance measurements^19,20^. Thus, it is plausible that these additional divacancy states could explain our observed decrease in carrier lifetime. However, pending a detailed description of the carrier capture and recombination mechanism, which requires further computational studies, this interpretation should be considered as being tentative.

**Figure 4:**
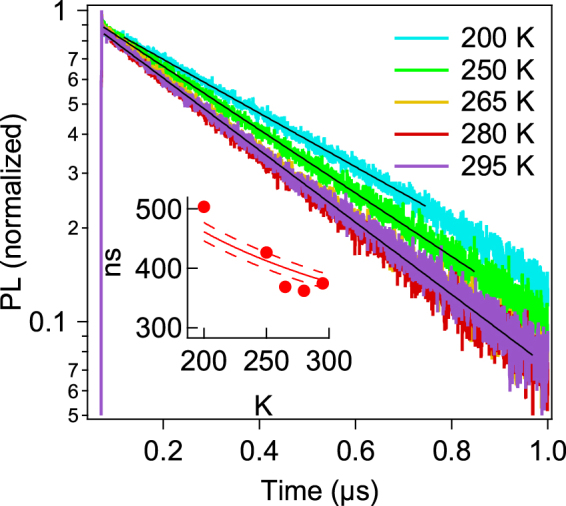
TRPL traces measured on the KF-treated E_g_ = 1.12 eV sample at 200–295 K with excitation at 1.94 eV and average power 12 µW. Black lines show single-exponential fits and the inset shows extracted lifetimes vs temperature fitted to Eq. (1).

To evaluate the metastable defect characteristics and their impact on CIGS solar cells, we analyze temperature-dependent TRPL data (Fig. 4) and use TCAD modeling to evaluate device performance. In contrast to reported results measured on CIGS solar cells fabricated by an industrial process^21^, no strong variation in lifetime is observed at temperatures near 300 K. In addition, the TRPL data for the high-efficiency solar cells studied here can be fully described with a single-exponential decay model. This leads us to conclude that minority-carrier trapping in shallow states is not a significant contributor to recombination in our samples. Single-exponential decays with lifetimes weakly decreasing at higher temperatures indicate Shockley-Read-Hall recombination dominated by deep defect states where the minority-carrier lifetime in the *p*-type material can be described by

1 $${{\boldsymbol{\tau }}}_{{\boldsymbol{SRH}}}={({{\boldsymbol{\sigma }}}_{{\boldsymbol{n}}}{{\boldsymbol{N}}}_{{\boldsymbol{t}}}{{\boldsymbol{v}}}_{{\boldsymbol{th}}})}^{-{\bf{1}}}$$

where *σ*_*n*_ is the electron capture cross section, *N*_*t*_ is the density of defect centers, and *v*_*th*_ is the thermal electron velocity given by ${v}_{{th}}=\sqrt{3{k}_{B}T/{m}_{n}}$, where *k*_*B*_ is the Boltzmann constant, *T* is temperature, *m*_*n*_ = 0.1 *m*_*e*_ is the effective mass^22^, and *m*_*e*_ is the electron mass. From the fit to (1) shown in the inset in Fig. 4, we find the product *σ*_*n*_ *N*_*t*_ to be in the range of 0.072 ± 0.002 cm^−1^.

**Figure 5:**
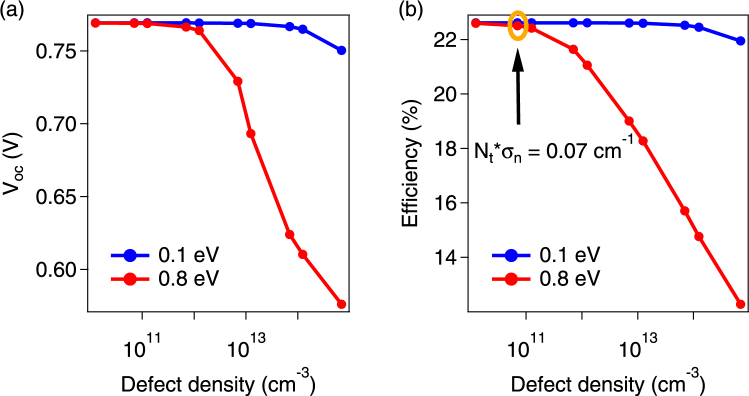
Simulated open-circuit voltage (**a**) and energy conversion efficiency (**b**) for CIGS as a function of the density of defects 0.1 eV and 0.8 eV above the VB.

Figure 5 shows *V*_*oc*_ and efficiency values calculated with TCAD for a range of densities of the two metastability-induced defects^12^ located at 0.1 eV and 0.8 eV above the VB, respectively. The defects were assumed to have a Gaussian energy distribution with a width *γ* = 0.05 eV, based on the FWHM determined experimentally (Fig. 1(b)). With TCAD, we analyze CIGS materials and devices with *E*_*g*_ = 1.12 eV where recombination in the bulk is presumed to be the dominant recombination pathway^23^. The capture cross section *σ*_*n*_ for electrons in the 0.8 eV defect and *σ*_*p*_ for holes in the 0.1 eV defect is assumed to be *σ*_*n,p*_ = 10^−12^ cm^2,^ ^24^. With this assumption, the product *σ*_*n*_ *N*_*t*_ = 0.07 cm^−1^ found above corresponds to a defect density of 7 × 10^10^ cm^−3^. The simulations with this defect density (Fig. 5(b)) result in a device efficiency of 22.5%, which is slightly higher than the experimental value of 20.6% reported for the same material in ref.^13^. The higher efficiency predicted by the model over the experimental value could be due to underestimation of the optical losses and overestimation of the current in the model, as well as the fact that lateral potential fluctuations, which can present a significant impact in device performance^13^, were not included in the model.

For *E*_*g*_ = 1.12 eV CIGS, the low defect density (corresponding to *σ*_*n*_ *N*_*t*_ = 0.07 cm^−1^) has a minimal impact on performance; however, commercial CIGS generally has a higher defect density as evident from significantly shorter carrier lifetimes^25^. Figure 5(b) explores the impact of higher defect density of each of these defects on device performance. The acceptor-type defect 0.1 eV above the valance band has a negligible impact on device efficiency for defects densities *N*_*d*_ < 10^14^ cm^−3^. The 0.8-eV defect, however, can have a much stronger impact on device performance (red lines in Fig. 5).

## Conclusions

We used excitation- and time-resolved photoluminescence spectroscopy to study defect states in high-efficiency CIGS absorbers. We found evidence of a state 125–155 meV above the valence band, which appears to be involved in the metastability behavior of this material. Exposure to sub-bandgap excitation induced an increase in acceptor concentration and a decrease in carrier lifetime, consistent with the appearance of an acceptor state and a recombination center predicted by the divacancy metastability model^12^. We find the product of the electron capture cross section and the density for the recombination center to be 0.072 ± 0.002 cm^−1^. Numerical simulations showed that the metastability-induced recombination center can have a strong negative effect on device performance when the defect density exceeds 10^12^ cm^−3^. Our findings provide guidance where further improvements are needed for CIGS solar cells.

## Methods

### Sample fabrication

Fabrication of the low-bandgap samples (*E*_*g*_ ≈ 1.1 eV) is described in ref^13^. This process was developed to fabricate solar cells with >20% power conversion efficiency. Ref.^13^ also describes device and material characteristics for the low-*E*_*g*_ samples studied here. One of the low-bandgap absorbers was given a KF postdeposition treatment immediately following the CIGS deposition^13,26^. For the high-bandgap (*E*_*g*_ = 1.50 eV) CIGS, the Ga/(In + Ga) ratio was increased to 0.8 and the substrate temperature during the second stage was about 615 °C. The high Ga absorber was finished into a device using the standard layers (CdS, ZnO bi-layer, grids) described elsewhere^27–29^.

### Photoluminescence characterization

Time- and spectrally resolved photoluminescence (TRPL and PL, respectively) were measured as described in Refs^13,30^. For optical measurements at variable temperature (5–300 K), we used a Cryostation (Montana Instruments) equipped with a cryogenic xyz translation stages. To directly excite the defect states, the excitation energy was tuned between 0.9 eV and *E*_*g*_ with a femtosecond optical parametric amplifier (Orpheus, Light Conversion). The repetition rate of the laser pulses was 1.1 MHz. Our TRPL spectrometer uses a 100 μm core diameter multimode optical fiber for excitation and signal collection^31^. The light was focused to the sample with an aspheric lens (New Focus 5724, numerical aperture NA 0.50), and the excitation spot diameter was approximately 0.3 mm. The same lens was used for PL signal collection. The optical-fiber-based setup enables a relatively large excitation spot size (important when low injection is needed) and efficient PL collection due to the relatively high NA of the lens.

### Simulations

First-principles calculations were performed with the VASP code^32,33^ using the GW approximation^34^ in a 64-atom supercell of CuInSe_2_ containing a *V*_Se_-*V*_Cu_ pair in the *q* = +1 charge state. A relatively dense 4 × 4 × 4 k-point mesh was used for Brillouin zone integrations to obtain the DOS induced by the defect. See refs^15,35^ for further computational details, including finite cell-size corrections for the energy of the defect state.

TCAD simulations were carried out with the Sentaurus Device package^13^. The model includes the bandgap profile determined from Auger electron spectroscopy (AES) data, lifetimes measured by TRPL, and doping density measured by capacitance-voltage^13^. More details about modeling are available in^13^. Absorption spectra used in the modeling were taken from^16^.

## References

[1] Jackson P (2016). Effects of heavy alkali elements in Cu(In,Ga)Se_2_ solar cells with efficiencies up to 22.6%. Phys. Status Solidi - Rapid Res. Lett.

[2] Green MA, Emery K, Hishikawa Y, Warta W, Dunlop ED (2016). Solar cell efficiency tables (version 48). Prog. Photovoltaics Res. Appl.

[3] Rau U, Schmitt M, Parisi J, Riedl W, Karg F (1998). Persistent photoconductivity in Cu(In,Ga)Se_2_ heterojunctions and thin films prepared by sequential deposition. Appl. Phys. Lett..

[4] Igalson M (1993). Photoconductivity of p–Type CuInSe_2_. Phys. Status Solidi.

[5] Meyer T, Schmidt M, Engelhardt F, Parisi J, Rau U (1999). A model for open circuit voltage relaxation in Cu(In,Ga)Se2 heterojunction solar cells. Eur. Phys. Journal. Appl. Phys.

[6] Sasala, R. A. & Sites, J. R. Time dependent voltage in CuInSe_2_ and CdTe solar cells. in *23rd IEEE Photovoltaic Specialists Conference* 543–548 10.1109/PVSC.1993.347036 (1993).

[7] Müller TCM (2013). Effect of light soaking on the electro– and photoluminescence of Cu(In,Ga)Se_2_ solar cells. Appl. Phys. Lett..

[8] Rau, U. *et al*. The inherent stability of Cu(In,Ga)Se2–based solar cells. in 2nd World Conference on Photovoltaic Solar Energy Conversion (eds. Schmid, J., Ossenbrink, H. A., Helm, P., Ehmann, H. & Dunlop, E. D.) 1, 428 (E. C. Joint Research Centre, 1998).

[9] Rau U, Schock HW (1999). Electronic properties of Cu(In,Ga)Se_2_ heterojunction solar cells–recent achievements, current understanding, and future challenges. Appl. Phys. A.

[10] Heath JT, Cohen JD, Shafarman WN (2003). Distinguishing metastable changes in bulk CIGS defect densities from interface effects. Thin Solid Films.

[11] Heath JT, Cohen JD, Shafarman WN (2004). Bulk and metastable defects in CuIn_1−x_Ga_x_Se_2_ thin films using drive-level capacitance profiling. J. Appl. Phys..

[12] Lany S, Zunger A (2006). Light- and bias-induced metastabilities in Cu(In,Ga)Se_2_ based solar cells caused by the (V_Se_−V_Cu_) vacancy complex. J. Appl. Phys..

[13] Jensen SA (2016). Beneficial effect of post-deposition treatment in high-efficiency Cu(In,Ga)Se_2_ solar cells through reduced potential fluctuations. J. Appl. Phys..

[14] Siebentritt S, Igalson M, Persson C, Lany S (2010). The electronic structure of chalcopyrites - bands, point defects and grain boundaries. Prog. Photovoltaics Res. Appl.

[15] Lany S (2013). Band-structure calculations for the 3d transition metal oxides in GW. Phys. Rev. B.

[16] Alonso MI, Garriga M, Durante Rincón CA, Hernández E, León M (2002). Optical functions of chalcopyrite CuGa_x_In_1−x_Se_2_ alloys. Appl. Phys. A Mater. Sci. Process.

[17] Metzger WK, Repins IL, Contreras MA (2008). Long lifetimes in high-efficiency Cu(In,Ga)Se_2_ solar cells. Appl. Phys. Lett..

[18] Pohl J, Albe K (2013). Intrinsic point defects in CuInSe_2_ and CuGaSe_2_ as seen via screened-exchange hybrid density functional theory. Phys. Rev. B.

[19] Heath JT, Cohen JD, Shafarman WN, Liao DX, Rockett AA (2002). Effect of Ga content on defect states in CuIn_1−x_Ga_x_Se_2_ photovoltaic devices. Appl. Phys. Lett..

[20] Hu X (2014). Investigation of deep-level defects in Cu(In,Ga)Se_2_ thin films by a steady-state photocapacitance method. J. Appl. Phys..

[21] Maiberg M, Hölscher T, Zahedi-Azad S, Fränzel W, Scheer R (2015). Investigation of long lifetimes in Cu(In,Ga)Se_2_ by time-resolved photoluminescence. Appl. Phys. Lett..

[22] Persson C (2008). Anisotropic hole-mass tensor of CuIn_1−x_Ga_x_(S,Se)_2_: Presence of free carriers narrows the energy gap. Appl. Phys. Lett..

[23] Li JV (2014). A recombination analysis of Cu(In,Ga)Se_2_ solar cells with low and high Ga compositions. Sol. Energy Mater. Sol. Cells.

[24] Gloeckler M, Fahrenbruch AL, Sites JR (2003). Numerical modeling of CIGS and CdTe solar cells: Setting the baseline. In Proceedings of the 3rd World Conference on Photovoltaic Energy Conversion.

[25] Mansfield LM (2015). Optoelectronic Investigation of Sb-Doped Cu(In,Ga)Se_2_. IEEE J. Photovoltaics.

[26] Rudmann D, Brémaud D, Zogg H, Tiwari AN (2005). Na incorporation into Cu(In,Ga)Se_2_ for high-efficiency flexible solar cells on polymer foils. J. Appl. Phys..

[27] Contreras, M. A. *et al*. High efficiency Cu(In,Ga)Se2-based solar cells: Processing of novel absorber structures. In *24th IEEE Photovoltaic Specialists Conference* 68–75 (1994).

[28] Contreras MA (1999). Progress toward 20% efficiency in Cu(In,Ga)Se_2_ polycrystalline thin-film solar cells. Prog. Photovoltaics Res. Appl.

[29] Contreras MA (2002). Optimization of CBD CdS process in high-efficiency Cu(In,Ga)Se_2_-based solar cells. Thin Solid Films.

[30] Repins IL (2015). Fiber-fed time-resolved photoluminescence for reduced process feedback time on thin-film photovoltaics. Rev. Sci. Instrum.

[31] Kuciauskas, D. *et al*. Optical-fiber-based, time-resolved photoluminescence spectrometer for thin-film absorber characterization and analysis of TRPL data for CdS/CdTe interface. In *38th IEEE Photovoltaic Specialists Conference* 1721–1726 (2012).

[32] Kresse G, Joubert D (1999). From ultrasoft pseudopotentials to the projector augmented-wave method. Phys. Rev. B.

[33] Shishkin M, Kresse G (2006). Implementation and performance of the frequency-dependent GW method within the PAW framework. Phys. Rev. B.

[34] Hedin L (1965). New method for calculating the one-particle Green’s function with application to the electron-gas-problem. Phys. Rev..

[35] Lany S, Zunger A (2010). Many-body GW calculation of the oxygen vacancy in ZnO. Phys. Rev. B.

